# Clinical guidelines for the management of weight during pregnancy: a qualitative evidence synthesis of practice recommendations across NHS Trusts in England

**DOI:** 10.1186/s12884-023-05343-9

**Published:** 2023-03-11

**Authors:** Lucy Goddard, Nerys M. Astbury, Richard J. McManus, Katherine Tucker, Jennifer MacLellan

**Affiliations:** Nuffield Department of Primary Care Health Sciences, University of Oxford, Oxford, UK

**Keywords:** Qualitative evidence synthesis, Pregnancy, Weight management care recommendations, Clinical guidelines, Birth Territory Theory

## Abstract

**Background:**

Women who enter pregnancy with a Body Mass Index above 30 kg/m^2^ face an increased risk of complications during pregnancy and birth. National and local practice recommendations in the UK exist to guide healthcare professionals in supporting women to manage their weight. Despite this, women report inconsistent and confusing advice and healthcare professionals report a lack of confidence and skill in providing evidence-based guidance. A qualitative evidence synthesis was conducted to examine how local clinical guidelines interpret national recommendations to deliver weight management care to people who are pregnant or in the postnatal period.

**Methods:**

A qualitative evidence synthesis of local NHS clinical practice guidelines in England was conducted. National Institute for Health and Care Excellence and Royal College of Obstetricians and Gynaecologists guidelines for weight management during pregnancy constructed the framework used for thematic synthesis. Data was interpreted within the embedded discourse of risk and the synthesis was informed by the Birth Territory Theory of Fahy and Parrat.

**Results:**

A representative sample of twenty-eight NHS Trusts provided guidelines that included weight management care recommendations. Local recommendations were largely reflective of national guidance. Consistent recommendations included obtaining a weight at booking and informing women of the risks associated with being obese during pregnancy. There was variation in the adoption of routine weighing practices and referral pathways were ambiguous. Three interpretive themes were constructed, exposing a disconnect between the risk dominated discourse evident in the local guidelines and the individualised, partnership approach emphasised in national level maternity policy.

**Conclusions:**

Local NHS weight management guidelines are rooted in a medical model rather than the model advocated in national maternity policy that promotes a partnership approach to care. This synthesis exposes the challenges faced by healthcare professionals and the experiences of pregnant women who are in receipt of weight management care. Future research should target the tools utilised by maternity care providers to achieve weight management care that harnesses a partnership approach empowering pregnant and postnatal people in their journey through motherhood.

**Supplementary Information:**

The online version contains supplementary material available at 10.1186/s12884-023-05343-9.

## Background

Less than half of women in the UK enter pregnancy with a Body Mass Index (BMI) below 30 kg/m^2^ [1]. Risks associated with a raised BMI include gestational diabetes, pre-eclampsia, postpartum haemorrhage, prolonged labour, birth defects and fetal macrosomia [2, 3]. Although well documented within the literature, a lack of awareness of the risks of obesity in pregnancy and challenges surrounding losing weight preclude many women from weight loss attempts prior to conception [4, 5].

Maternity health care professionals indicate a lack of confidence, knowledge and skill as significant barriers to delivering effective and supportive weight management care [6]. As a result, women report inconsistent advice, confusion over the information they do receive and a lack of support from healthcare professionals despite wanting weight management advice [7–10]. This leads women to seek information from other sources, and can induce anxiety around weight gain [9, 11].

The UK National Institute for Health and Care Excellence (NICE) and Royal College of Obstetricians and Gynaecologists (RCOG) have produced guidelines for weight management before, during and after pregnancy [12, 13]. They provide recommendations for maternity practitioners in how to deliver safe care for women classified as obese (BMI ≥ 30 kg/m^2^) at the start of pregnancy. Based on the best available research evidence and expert consensus, these national guidelines are interpreted by NHS Trusts in England to fit the local context, population, resources and infrastructure. They turn best practice recommendations into practical actions at the interface of maternity care delivery, supporting practitioners to support women and birthing people in optimum weight management.

The aim of this review was to analyse how local NHS clinical guidelines for weight management during pregnancy and the postnatal period interpret national guidance, synthesising findings to extend interpretation of observed variations between and within national and local guidelines.

## Methods

### Design

We performed a qualitative evidence synthesis of local NHS clinical practice guidelines using the framework analysis approach of Gale et al. (2013) and synthesis method of Thomas and Harden (2008) [14, 15]. Analytical rigour and transparency were ensured through careful documentation of the synthesis process described here.

### Search method

We approached a purposive sample of NHS Trusts across England. A sample of at least 28 Trusts was selected pragmatically by the principle researcher (LG) aiming to ensure representation of Trusts from the seven NHS regions in order to provide adequate variation of the pre-specified attributes [16]. These attributes were considered to potentially influence the results and included rural/ urban setting, catchment population size, number of births per year and university/ teaching status. Guidelines were downloaded from their website or an application was made through a Freedom of Information process (email or online form) between 1^st^ April and 1^st^ June 2021. In the case of non-responders, a Trust within the same region of similar size and university status was contacted. Upon receipt of each guideline, the associated Trust was assigned a unique sequential code to ensure anonymity.

### Quality appraisal

The purpose of this review was not to judge the quality of the existing guidelines but rather sought to present what is currently recommended and examine the content of the guideline. Therefore, no quality appraisal was performed.

### Data abstraction

Narrative sections of the localised guidelines were extracted and entered verbatim into NVivo for coding (QSR International Pty Ltd. (2020) NVivo (released in March 2020)).

### Synthesis

Narrative data were coded into a deductive framework of 18 pre-defined categories, constructed from the recommendations of the NICE Weight management in pregnancy guideline and the RCOG Care of Women with Obesity in Pregnancy guideline by the principle researcher (LG) [12, 13] (Table 1). Descriptive memos for each category supported consistency in coding. Each pre-defined category, and the coded text within it, was extracted and imported into Excel (Microsoft Excel 2016) to generate a matrix, enhancing visualisation for data comparison and interpretation. Cells were tagged with direct quotes or important interpretations, in order to capture nuances and ambiguity between guidelines. This led to collapse and refinement of categories into broad descriptive themes. Reflexivity was practiced throughout the data synthesis, supported by discussions with the full research team. Interpretations were informed by risk discourse and the Birth territory Theory, to synthesise the descriptive themes into three analytical themes that structure the presentation of our synthesis findings [17, 18].

**Table 1:** Framework based on pre-specific categories constructed from NICE and RCOG guidelines

NICE/ RCOG recommendation (category)	Description and instruction
Address any concerns about physical activity in pregnancy	NICE: "Advise that moderate-intensity physical activity will not harm her or her unborn child."
	This includes reassuring women about the safety of physical activity in pregnancy
Addressing a woman's concerns	NICE: Be "…sensitive to any concerns she may have about her weight."
	Any discussion on the sensitive manner in which conversations about weight should be had and the importance of communication between the woman and healthcare professionals
Advice on benefits of healthy diet and physical activity	NICE: "Advise that a healthy diet and being physically active will benefit both the woman and her unborn child during pregnancy and will also help her to achieve a healthy weight after giving birth."
	Discussions with the healthcare professional around healthy eating and being physically active that is not specific. Code as ‘Specific dietary or physical activity advice’ if specific advice is given
Behaviour change advice	“Evidence-based behaviour change advice includes: understanding the short, medium and longer-term consequences of women's health-related behaviour helping women to feel positive about the benefits of health-enhancing behaviours and changing their behaviours recognising how women's social contexts and relationships may affect their behaviour helping plan women's changes in terms of easy steps over time identifying and planning situations that might undermine the changes women are trying to make"
	Only code this if it does not fit into any other code. For example, if they are provided with individualised advice, code only as ‘Practical and tailored information’. This is because this code describes the way in which advice (within the other codes) may be provided
Breastfeeding	NICE: "Midwives and other health professionals should encourage women to breastfeed. They should reassure them that a healthy diet and regular, moderate-intensity physical activity and gradual weight loss will not adversely affect the ability to breastfeed or the quantity or quality of breast milk."
	RCOG: "Obesity is associated with low breastfeeding initiation and maintenance rates. Women with a booking BMI 30 kg/m2 or greater should receive appropriate specialist advice and support."
Dieting or weight loss in pregnancy	NICE: "Dieting during pregnancy is not recommended as it may harm the health of the unborn child."
	RCOG: "Anti-obesity or weight loss drugs are not recommended for use in pregnancy."
	Any recommendation that refers to dietary programmes/ diets that promote weight loss
Discuss her current eating habits and physical activity levels	NICE: "At the earliest opportunity, for example, during a pregnant woman's first visit to a health professional, discuss her eating habits and how physically active she is. Find out if she has any concerns about diet and the amount of physical activity she does and try to address them."
Dispelling myths	NICE: "Dispel any myths about what and how much to eat during pregnancy. For example, advise that there is no need to 'eat for two' or to drink full-fat milk. Explain that energy needs do not change in the first 6 months of pregnancy and increase only slightly in the last 3 months (and then only by around 200 cal per day)."
Explaining risks	NICE: "Explain to women with a BMI of 30 or more at the booking appointment how this poses a risk, both to their health and the health of the unborn child. Explain that they should not try to reduce this risk by dieting while pregnant and that the risk will be managed by the health professionals caring for them during their pregnancy."
	Do not code if risks are only stated at the beginning of the guideline (as part of the introduction). It should be clear whether risks are discussed with the woman
Height and weight at the first contact	NICE: "Measure weight and height at the first contact with the pregnant woman" "Weight, height and BMI should be recorded in notes, the woman's hand-held record and the patient information system"
Not within NICE or RCOG recommendations	Anything that is related to weight management that is not referred to by NICE or RCOG guidelines
Practical and tailored information	NICE: "Offer practical and tailored information. This includes advice on how to use Healthy Start vouchers to increase the fruit and vegetable intake of those eligible for the Healthy Start scheme (women under 18 years and those who are receiving benefit payments)."
	Any guideline that indicates providing personalised or tailored advice based on the individual woman. If this is coded, check for code “Discuss her current eating habits and physical activity levels”
	If women are referred to a dietician or other programme it is likely they will receive personalised advice however for the purpose of examining the guideline only and therefore, first line of care, do not code if not explicitly described that advice is personalised or tailored
Recommended weight gain ranges	NICE: "Many pregnant women ask health professionals for advice on what constitutes appropriate weight gain during pregnancy. However, there are no evidence-based UK guidelines on recommended weight-gain ranges during pregnancy.”
	RCOG: "There is a lack of consensus on optimal gestational weight gain. Until further evidence is available, a focus on a healthy diet may be more applicable than prescribed weight gain targets."
	Code if weight gain thresholds are given (and which ones, i.e. IOM) even if the lack of evidence on weight gain ranges is acknowledged
Referrals	NICE "Offer women with a BMI of 30 or more at the booking appointment a referral to a dietitian or appropriately trained health professional for assessment and personalised advice on healthy eating and how to be physically active."
	Does not include obstetric or anaesthetic referrals for risk assessment or birth planning. Only referrals relating to weight management
	Code even if guideline is not clear about who the referral is for or who/ where they are being referred
	Code if weight management programmes are highlighted, for example, Slimming World, even if there isn’t a formal referral process described
Signposting to reputable sources of information	NICE "Reputable sources of information and advice about diet and physical activity for women before, during and after pregnancy include: The Department of Health's 'The pregnancy book' and 'Birth to five' and the NHS eat well website." "Advise her to seek information and advice on diet and activity from a reputable source"
	Code if women are provided with a Trust leaflet/ signposted to Trust website to access content on weight management during pregnancy but do not code if these resources only detail obstetric or anaesthetic risks
Specific dietary or physical activity advice	NICE: Specific dietary advice, e.g. eat fibre rich foods, eat at least 5 portions of fruit and vegetables each day, avoid increasing calorie intake. Specific physical activity advice e.g. 150 min of exercise per week
Weighing women	NICE: "Do not weigh women repeatedly during pregnancy as a matter of routine. Only weigh again if clinical management can be influenced or if nutrition is a concern"
	RCOG: “For women with obesity in pregnancy, consideration should be given to reweighing women during the third trimester to allow appropriate plans to be made for equipment and personnel required during labour and birth”
	Code if women are weighed throughout pregnancy with information on recommended gestations
	Code if recommendation is risk assessment weight in the third trimester as this is a weight that would influence clinical management. However, it is important to note, this is not for weight management purposes. For example, it is not for the purpose of starting a conversation with the woman about her diet and physical activity levels
Weight management postpartum	NICE: "Encourage them to lose weight after pregnancy."
	RCOG: "Women should be supported to lose weight postpartum and offered referral to weight management services where these are available."
	Code both those that recommend providing healthy lifestyle advice and those that signpost women to other healthcare professionals/ weight management services

### Theoretical framework

Identification of a theoretical underpinning is vital in midwifery research to tackle and explore complex phenomena, such as weight management, as it ensures findings consider different perspectives; essential to improve care practices and thus, women’s experiences [19]. Birth Territory Theory (BTT), chosen as the theoretical framework here, concerns the balance of power in the birth room and how maternity professionals can support and work in partnership with women [17, 18]. ‘Midwifery guardianship’, the theories key concept, exemplifies integrative power described as when the power between all present during care is equal and the shared, desired outcome is for the woman to feel confident and empowered. In contrast, disintegrative power disables the woman’s confidence in her abilities to achieve a positive pregnancy and birth experience and can induce the woman to become passive in her own care. Disintegrative power, enacted through medical surveillance, draws closely on the concept of risk embedded in maternity services [20, 21].

The Birth Territory theory and the concepts within it, reflect the ambition of UK maternity policy to listen to, understand and work in partnership with each individual woman to ensure a positive pregnancy and birth experience whilst minimising poor outcomes. Thus, the BTT was appropriate to provide an in-depth interpretation of the synthesis findings in the context of current midwifery practice.

## Results

A total of 29 hospital NHS Trusts were contacted and 28 guidelines received (Fig. 1, Additional file 1). Nine were classified as rural or remote, and 14 as University or teaching hospitals, with five classified as both rural or remote with University or teaching status. The number of hospitals within individual Trusts ranged from one to eight, with number of births for the year 2019–2020 ranging from 955 to 14,270 (Table 2) [22–24]. All the guidelines received classified women with obesity as having a BMI ≥ 30 kg/m^2^, with the exception of one guideline (Guideline C) classifying women as obese with a BMI ≥ 35 kg/m^2^, for the purpose of the guideline.

**Table 2:** Characteristics of NHS Trusts in England who provided guidelines

Code allocated to NHS Trust	Number of births per year^a^	Rural or remote^b^	Teaching/ University status	No. main hospital sites within Trust	Name of guideline
B	955	Remote	No	1	Standard Operational Procedure for the Management of Pregnancy Women whose Body Mass Index is ≥ 30 kg/m^2^
H	1295	Remote	No	1	Obesity in Pregnancy guideline
Y	1650	Remote	No	1	Guideline for the Management of obesity during pregnancy
AA	1925	No	No	1	Obesity in pregnancy birth and postnatal period
C	1960	Rural	No	1	Clinical guideline for the management of Obesity in Pregnancy
L	3775	No	Yes	1	Obesity in pregnancy, labour and puerperium
X	5840	No	No	1	The management of obesity in pregnancy
K	2580	No	Yes	2	Care of Pregnant Women with a Raised BMI ≥ 30 kg/m^2^
Q	3440	No	Yes	2	BMI Management in Pregnancy
E	4065	No	No	2	Raised Body Mass Index in Pregnancy
O	4270	Rural	Yes	2	Obesity: Management of Pregnant Women with a Raised BMI at Initial Consultation
BB	5125	Rural	Yes	2	Assessing and managing extremes of BMI in pregnancy
F	1495	No	No	3	Obesity in Maternity Care Guideline
A	2825	Remote	Yes	3	Obesity in Pregnancy
G	3805	No	No	3	Management of Obesity in Pregnancy Guideline
S	4010	Rural	Yes	3	Increased Body Mass Index (BMI) in Pregnancy, Labour and Post Delivery Clinical Guideline
J	4955	No	No	3	Management of obese women in pregnancy
U	5585	No	No	3	Obese Pregnant Women
W	5910	No	No	3	Management of Women with Obesity in Pregnancy
M	6310	No	No	3	Obesity in pregnancy
N	9030	No	No	3	Care of women with obesity in pregnancy
R	5230	No	Yes	4	BMI: Optimal weight for pregnancy and childbirth. Guidelines for women with BMI > 30 kg/m^2^
Z	14,270	No	Yes	4	Obesity in Pregnancy
T	8960	No	Yes	5	Obesity in Maternity
I	6007	No	Yes	6	Raised BMI during pregnancy
D	4820 4540 Figures from two merged Trusts	No	Yes	7	Obesity in Pregnancy
P	4420	Remote	Yes	8	Obesity guidelines
V	7640	No	Yes	1	Management of a pregnancy for women with a BMI > 30 kg/m^2^
**Total = 28**	N/a	9 rural/ remote	14 University/ teaching hospitals	N/a	N/a

^a^SOURCE: NHS Digital 2019/20

^b^Rural is classed as the top 10% of Trusts with the highest proportion of patients living in rural areas in 2018/19, calculated from Hospital Episode Statistics. Remote is classed as those with 'unavoidable small hospitals' (SOURCE: Nuffield Trust)

The synthesis of local clinical guideline interpretations of national guidance to deliver weight management care to people who are pregnant or in the postnatal period identified three core themes:

1. *Recommendations at odds with a partnership approach to care*
2. *Advocating surveillance – to what end?*
3. *Discretionary and ambiguous pathways*

### Recommendations at odds with a partnership approach to care

Healthcare professionals are advised in national guidance to discuss weight in a sensitive manner and allow opportunities for a woman to raise the concerns she may have about her lifestyle and the ways to address these [12, 13]. The local guidelines reflected this in varying degrees in their weight management recommendations. Ten emphasised the importance of sensitive and respectful conversations around weight where the language used was an important contributor to achieve a good relationship with the woman that laid the foundation for an equalised partnership (Guideline H, I, L, M, S, X, Y, Z, AA, BB). Seven guidelines further emphasised the importance of this to empower women (Guideline S, Z, AA, BB, H, M), to encourage active engagement in care (Guideline AA, BB, H, M, Z), and to promote a positive pregnancy and birth experience (Guidelines L, H). Three guidelines highlighted the sensitive nature of conversations around weight stigma and the lower self-esteem that may be experienced by women of a higher weight (Guideline L, M and I). An awareness of weight stigma and lower self-esteem was not addressed in twenty-five guidelines. A clinical approach was advocated in five guidelines where additional mental health screening was recommended (Guideline J, N, P, X, Z). This reflects RCOG recommendations that as women with a BMI above 30 kg/m^2^ are at an increased risk of mental health problems, they should be screened for these during pregnancy, without necessarily exploring the source of the mental distress [13].

Twenty-seven of the 28 guidelines recommended healthcare professionals discuss the risks associated with obesity during pregnancy with some recommending to explain “*the ways in which they [the risks] may be minimised*” or management strategies to reduce the risks (Guidelines A, C, D, E, F, L, Q, U, Z). Guidelines mostly provided a list of possible risks for healthcare professionals to discuss, with one guideline (Guideline N) advising a sticky label, listing the risks, be placed in the women’s hand-held notes. 5/28 guidelines provided risk calculations alongside at least one complication (Guidelines M, O, V, X, Y). No guideline offered a risk assessment framework to help healthcare professionals frame risk, or guidance on how risks may be minimised. Guideline H recommended that women should be advised of the risks but be reassured that most women with a raised BMI have straightforward pregnancies and healthy babies. This is unlike the general tone around risk observed in the majority of guidelines but paid attention to how risk information is translated by a woman who may already be feeling vulnerable. Conversely, it could cause confusion in why risk is discussed if most women experience no complications or alternatively, provide false reassurance regarding their potential for poorer outcomes [25].

Advocating a healthy lifestyle and the provision of practical advice on how to improve dietary intake and activity levels are recommended in national guidelines [12, 13]. Informing women of the benefits of adopting health behaviours during pregnancy was recommended in most local guidelines (26/28) but were limited in how they could work with women to achieve this. Seven guidelines offered brief recommendations on what a diet should consist of and recommendations for physical activity but overall information on how to translate this into the reality of women’s lives was not evident (Guideline F, J, K, M, X, Y, Z).

According to national guidance, healthcare professionals should tailor weight management advice to the individual needs of birthing people, enlisting behaviour change knowledge and techniques to provide an individualised approach to care [12]. At a local level, three guidelines mirrored this more collaborative approach (Guideline E, F, G). They recommended healthcare professionals to initiate questions about the woman’s own personal lifestyle, offering a woman an opportunity to frame their experience in a non-judgemental space; exemplifying midwifery guardianship. Three guidelines recommended addressing women’s concerns around physical activity (Guideline F, I, M) or weight (Guideline F, M) but no guidelines made recommendations to address women’s concerns specifically about her diet. Understanding the woman and providing an opportunity to listen to her concerns is central to ensuring equality in care. Guideline G described behavioural techniques such as goal setting to support women to makes changes and expressed that “*every contact with women during pregnancy…is an opportunity for tailored health promotion*”. Practical steps to identify barriers to behaviour change with women and make plans to overcome them were not clear in any guideline.

Twenty-two guidelines advised midwives to signpost women to reputable sources about healthy lifestyles during pregnancy. As stated in guidance, all women should be provided with accurate and *accessible* information but only one guideline explicitly identified leaflets that were provided in different languages (Guideline S). It was not evident in other guidelines that there was leaflet provision for those whose first language was not English. If supported with good communication, providing leaflets can be useful in providing women with information and tools to engage in healthy behaviours during pregnancy. However, if it is not reinforced with a conversation and incorporated into the woman’s individual context, it risks being discarded [26].

### Advocating surveillance – to what end?

National guidance recommends that an accurate height and weight should be obtained at the first antenatal contact and a further weight in the third trimester should be considered to plan for appropriate equipment and personnel for birth [12, 13]. Additional weight measurements should only be taken when “*clinical management can be influenced or if nutrition is of concern”.* All 28 guidelines recommended a baseline BMI to be calculated at the booking (27/28) or first scan (1/28) appointment. An initial weight measurement is part of the risk assessment to determine the appropriate clinical pathway for the woman and her pregnancy as women with a higher BMI have poorer outcomes and therefore should have increased surveillance [13]. The majority of guidelines (24/28) recommended further weight measurements with 13 aligning with national guidance to re-weigh for risk assessment purposes or if nutrition was a concern (Guidelines E, F, G, J, M, N, O, Q, V, W, Z, AA, BB). Eleven recommended healthcare professionals to monitor weight throughout pregnancy (Guideline K, R, H, I, L, P, S, T, U, X, Y). The purpose of re-weighing was clear in only two guidelines to support weight management (Guideline K and R). Women need clear and consistent messaging to feel encouraged and supported. Re-weighing with no given rationale can take away a woman’s sense of control especially as the guidance on how to act on a weight measurement was not clear in any guideline. Monitoring weight throughout pregnancy strongly speaks to the idea that pregnancy is an opportunity for increased surveillance to bring the body back to the “norm” and to identify when further surveillance or intervention is needed in order to guarantee safety as defined by the medical system. Weighing women for the purpose of clinical management, such as for drug calculations, is an example of this and has an important role in ensuring the woman receives appropriate and safe care. However, weighing women with no rationale or no subsequent guidance could be interpreted as controlling the pregnancy but to no clear advantage to the woman or the healthcare professional. For some women, weighing can induce feelings of shame and guilt and this needs considering within the recommendation when there is no clear pathway following a measurement.

National guidance does not provide weight gain thresholds for pregnancy because it concludes that there is no evidence or a lack of consensus on optimal gestational weight gain [12, 13]. Whilst 15 guidelines acknowledged this, eight guidelines recommended weight gain thresholds (Guideline I, M, Q, AA, J, R, X, Y). These were based either on the Institute of Medicine guidelines or on recommendations of which the source was unclear [27]. Current maternity care in the UK sits within a discourse of risk that perhaps explains the advocacy or attempts to adopt weight gain thresholds but we do not have the evidence in which to determine what a healthy gain looks like. This non-conclusive evidence results in recommendations lacking information about what to do should a woman fall above or below thresholds. This challenges the healthcare professional’s ability to support the woman effectively, thus, putting at risk the trusting relationship.

### Discretionary and ambiguous pathways

National guidance recommends a referral should be offered to women with a BMI of 30 kg/m^2^ or above at booking and then again in the postnatal period [12, 13]. A dietician or appropriately trained healthcare professional can then perform an assessment and provide personalised advice on a healthy lifestyle. Referrals for additional weight management support were suggested (21/28) but clear eligibility criteria and how to initiate the referral were frequently missing or unclear. Table 3 displays a breakdown of the suggested places for referral. Referrals were sometimes at the healthcare professional’s discretion to consider whether it was necessary and was dependent on resources or availability of services in the area. Referral pathways could have an important role in offering women an opportunity for more specialised or individualised care regarding her weight that may not be possible in routine appointments. Only Guideline R had a clear referral process where attendance at a specific workshop was requested via the midwife as early as possible. An additional referral was offered to women with a BMI above 25 to funded weight management services that included either one-to-one telephone support with a specialist midwife or support from a dietician via an App, depending on the area.

**Table 3:** Types of referrals suggested in the guidelines

Referral type	Number of Trusts	NHS Trust Code
Dietician/ dietetic service	15	B, E, F, H, I, K, L, N, Q, R, X, Y, Z, AA, BB
Weight management programme (unspecified)	3	G, A, AA
Weight management programme offered within the Trust^a^	8	D, F, K, L, M, O, Q, R,
Weight management programme offered in the community^b^	5	K, O, P, R, U

^a^Specified antenatal classes, workshops, specialist midwife follow-up or similar

^b^Community programmes including Slimming World (*n* = 3) or Weight Watchers (*n* = 1)

National guidance recommends that healthy lifestyle messages should be reinforced in the postnatal period and women should be advised to seek further support to manage weight or dietetic advice [12, 13]. This was recommended in 23 guidelines. Pregnancy is a life event for the woman and her family that continues beyond the remit of maternity service provision. The transition in services after birth is a tentative period as intervention, advice and information is often suspended or orientated toward the baby meaning women can feel a sudden loss of support and advice. Guideline I recommended that healthcare professionals write to the GP to consider measures to help with weight reduction. This transfer and sharing of information with the woman’s GP could provide a useful support link when women no longer fall within the scope of maternity services. Clear referral pathways after birth are essential in upholding the important health messaging established during pregnancy that are fundamental in determining the potential course of subsequent pregnancies. Breastfeeding was advocated, specifically in relation to supporting weight management, in four guidelines. (Guideline A, D, G, R).

## Discussion

### Key findings

While local recommendations were largely reflective of national guidance, our synthesis has exposed a disconnect between the partnership approach emphasised in national maternity policy, and the risk dominated discourse of local practice recommendations for weight management during pregnancy. The most consistent recommendations were to obtain a weight measurement at booking, inform women of the benefits of a healthy lifestyle and of the risks associated with being obese during pregnancy. Specific, practical advice about how to achieve a healthy lifestyle was lacking, there was variation in the adoption of routine weighing practices and referral pathways were ambiguous.

The Birth Territory Theory challenges the focus on surveillance and risk observed in the majority of guideline recommendations. In contrast but in alignment with the BTT, few guidelines promoted or facilitated an individualised or partnership approach; a characteristic of midwifery guardianship that displays an awareness of the sensitive and emotive nature of weight management conversations.

### Existing literature

Healthcare professionals face significant personal and contextual barriers to having conversations about weight during pregnancy [28–30]. Barriers include a lack of knowledge, skill, confidence and a fear of stigmatising women who enter pregnancy with obesity. The focus on risk, absence of clear instruction on providing behaviour change advice, unclear pathways and omission of practical lifestyle advice on how to work with women to manage weight that was observed in the local guidelines offers explanation to why such barriers may exist. Weight management advice is often sought by women but insensitive counselling around weight and in particular, risk, and the medicalisation of these pregnancies can result in depersonalisation of care and feelings of shame and guilt [6, 8, 31]. Creating a non-judgemental space, where a woman feels safe to discuss her concerns with the healthcare professional is fundamental within conversations about weight that do not induce weight stigma. However, local guidelines appear to fail healthcare professionals in advocating midwifery guardianship.

Local clinical guidelines derive from a medical perspective in which healthcare professionals dictate the course of clinical management, intervention and treatment based on the best available evidence. However, weight management is complex and incorporates biological, psychological, environmental and social factors, and the guidelines examined often overlooked these factors and were orientated around risk and surveillance. Focus on risk disempowers women and elevates anxiety [26]. During risk conversations, women can feel judged for their weight that can affect her decisions during pregnancy and if complications arise, made to feel they are to blame [25]. Pregnant women understand risk in a context specific way dependent on their previous experiences and socio-cultural factors emphasising the importance of individual assessment and framing of risk around the woman’s cultural and social context to support her sense making [32]. Women want constructive advice to follow new information about her risk and the omission of this detail creates challenges for healthcare professionals to ameliorate the anxieties created by risk counselling [33]. Clinical guidelines can support the complex interplay between scientific evidence, healthcare provider’s expertise and the woman’s preferences by considering how each recommendation and the evaluated outcome may support, or inhibit, personalised and sensitive conversations that would instigate a partnership approach to care [34].

### Implications for research and practice

Reconceptualising weight management care to align with the partnership approach that enacts midwifery guardianship is warranted. Whilst we work in a medical system that utilises clinical guidelines to inform healthcare professionals, recommendations must consider how a woman’s social, environmental and psychological context can be considered in order to reduce weight stigmatisation, increase her choice and empower her. To achieve the partnership approach that maternity care policy advocates, the manner in which weight conversations in pregnancy are had, the subsequent communication of practical, individualised advice as well as the dominant risk-focus of the guidelines must be re-examined.

Implementing evidence-based recommendations must be complemented by equipping healthcare professionals with specific training to increase skills, knowledge and confidence, frequently identified as barriers to providing supportive weight management care. This includes offering professionals support in communicating risk about weight in a way that the woman can make sense of considering her values, beliefs and preferences to dissipate feelings of anxiety or guilt. Co-designing clinical guidelines with women who have experienced pregnancy with obesity or excessive gestational weight gain could be fundamental in the design of tools we use to deliver weight management care.

### Strengths and limitations

To our knowledge, this is the first study to use a theoretical framework to understand weight management guidelines that are used across different NHS Trusts in England. Adding to existing evidence surrounding weight management care provision during pregnancy, this study helpfully adds a new perspective that challenges current practices by highlighting the disconnect between the ambition of maternity policy and guideline recommendations. It has also provided some context for the barriers faced by healthcare professionals as well as the experiences of pregnant women; providing momentum for ongoing inspection of national and local clinical guidelines that may hinder progression towards supportive, collaborative weight management care that empowers women.

A purposive sample generated a well-represented national picture of guidelines from all seven NHS regions in England. Maternity units in Scotland, Wales and Northern Ireland were not included due to the differences in NHS or non-NHS healthcare structures, which would have made comparability with NICE and RCOG guidelines difficult. Therefore, a study comparing local guidelines beyond England may be warranted.

Using the NICE and RCOG guidelines as a deductive analysis framework was useful to ensure we explored all aspects of weight management care. However, we did not include associated guidelines in our framework such as ‘Maternal and child nutrition’ [PH11], ‘Antenatal care’ [NG201] or ‘Postnatal care’ [NG194] guidelines [35–37]. It is important to acknowledge that they may provide additional detail on the care women receive. However, the focus of this synthesis was weight management and therefore, to ensure the study had a clear focus on this, other guidelines were not used in the framework for analysis.

## Conclusion

Our guideline synthesis has shown that locally interpreted weight management guidelines are rooted in a risk discourse, rather than a partnership approach that is advocated in maternity policy. This disconnect could explain the rifts in weight management care delivery that is evidenced in existing literature. Although some consistent recommendations existed and were reflective of national guidance, ambiguous care pathways and unclear recommendations prevailed that would hinder a healthcare professionals ability to translate these into practice. Future research should address the tools in which maternity healthcare providers use to provide weight management care and a revaluation of the system in which they work in order to achieve the ambition for all pregnant and postnatal people to receive sensitive, personalised and safe weight management care.

## Supplementary Information

**Additional file 1:** **Figure 1.** A map of NHS Trusts in England contacted for guidelines. Map created with ZeeMaps (www.zeemaps.com). 

## Data Availability

The datasets used and/or analysed during the current study are available from the corresponding author on reasonable request.

## Abbreviations

BMI: Body Mass Index
NICE: National Institute for Health and Care Excellence
GWG: Gestational Weight Gain
RCOG: Royal College of Obstetricians and Gynaecologists
NHS: National Health Service
BTT: Birth Territory Theory
IOM: Institute of Medicine
