# Supplementary material for: Clinical guidelines for the management of weight during pregnancy: a qualitative evidence synthesis of practice recommendations across NHS Trusts in England
Source: BMC Pregnancy Childbirth. 2023 Mar 11;23:164. doi: 10.1186/s12884-023-05343-9 (PMC10007759; doi:10.1186/s12884-023-05343-9)
Supplement: Supplementary file 1 — Additional file 1: Figure 1. A map of NHS Trusts in England contacted for guidelines. Map created with ZeeMaps (www.zeemaps.com). [file 12884_2023_5343_MOESM1_ESM.docx]

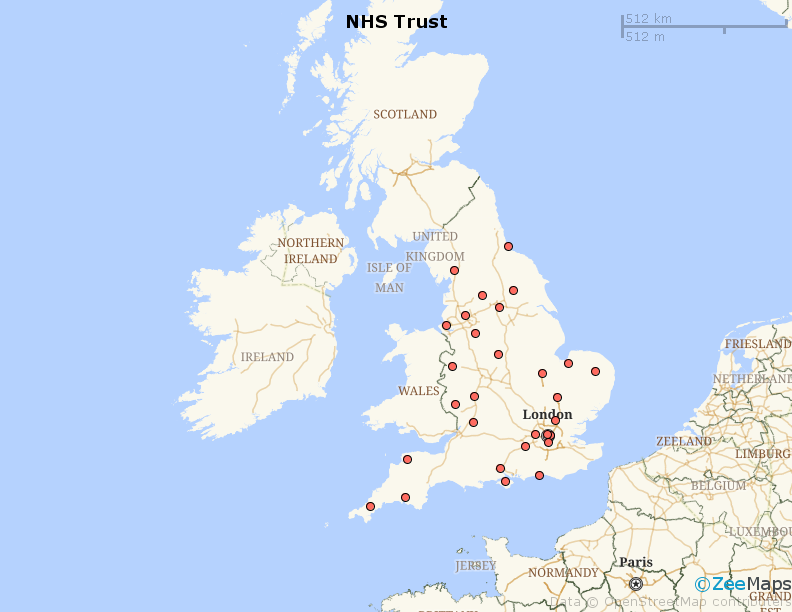


Figure 1 A map of NHS Trusts in England contacted for guidelines. Map created with ZeeMaps ([www.zeemaps.com](http://www.zeemaps.com)).
